# Three-Dimensional Structure of Biofilm Formed on Glass Surfaces Revealed Using Scanning Ion Conductance Microscopy Combined with Confocal Laser Scanning Microscopy

**DOI:** 10.3390/microorganisms13081779

**Published:** 2025-07-30

**Authors:** Nobumitsu Hirai, Yuhei Miwa, Shunta Hattori, Hideyuki Kanematsu, Akiko Ogawa, Futoshi Iwata

**Affiliations:** 1 Department of Chemistry and Biochemistry, National Institute of Technology (KOSEN), Suzuka College, Shiroko-Cho, Suzuka 510-0294, Japanogawa@chem.suzuka-ct.ac.jp (A.O.); 2 Department of Mechanical Engineering, Faculty of Engineering, Shizuoka University, 3-5-1 Johoku, Chuo-ku, Hamamatsu 432-8561, Japaniwata.futoshi@shizuoka.ac.jp (F.I.); 3 Joint Research Center Between Academia and Industries, National Institute of Technology (KOSEN), Suzuka College, Shiroko-Cho, Suzuka 510-0294, Japan; h.kanematsu@mat.eng.osaka-u.ac.jp

**Keywords:** biofilm, scanning ion conductive microscope, confocal laser scanning microscope, *Aliivibrio fischeri*, in situ observation

## Abstract

Biofilms cause a variety of problems, such as food spoilage, food poisoning, infection, tooth decay, periodontal disease, and metal corrosion, so knowledge on biofilm prevention and removal is important. A detailed observation of the three-dimensional structure of biofilms on the nanoscale is expected to provide insight into this. In this study, we report on the successful in situ nanoscale observations of a marine bacterial biofilm on glass in phosphate buffer solution (PBS) using both scanning ion conductance microscopy (SICM) and confocal laser scanning microscopy (CLSM) over the same area. By observing the same area by SICM and CLSM, we were able to clarify the three-dimensional morphology of the biofilm, the arrangement of bacteria within the biofilm, and the difference in local ion conductivity within the biofilm simultaneously, which could not be achieved by observation using a microscope alone.

## 1. Introduction

**Figure 1:**
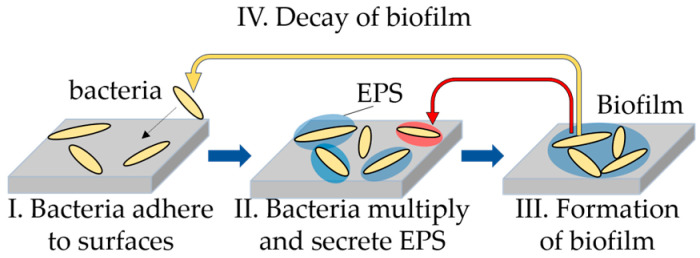
Schematic diagram of biofilm formation.

Biofilms exist wherever there is water and are widely found in nature. The process of biofilm formation is shown in Figure 1. First, bacteria attach to the surface of a material, and then the biofilm-forming bacteria grow and produce extracellular polymeric substances (EPSs), which act as a barrier to protect the bacteria inside from environmental changes and chemicals. This is thought to aid in the formation of a dense, closed colony and maintain homeostasis. Biofilms consist of 70–90% water, bacteria, and EPSs, which in turn comprise polysaccharides, proteins, extracellular DNA (eDNA), and lipids [1]. Finally, the biofilm matures, and some of the bacteria that formed the biofilm are released into the water.

Bacteria that live in biofilms interact with the materials. Specifically, they cause microbial-influenced corrosion when they form on metal surfaces; tooth decay when they form on teeth; infections when they form in hospitals; and food spoilage or food poisoning when they form on food surfaces. Because biofilms cause a variety of problems, knowledge of how to prevent and remove them is extremely important, but currently, only symptomatic approaches exist. It is known that bacterial inactivation on a surface and biofilm formation can be influenced by the properties of the surface. The surface characteristics of glass have an influence on the inactivation of *E. coli* O157, *S. Typhimurium*, and *L. monocytogenes* via ClO_2_ gas treatment [2]. The vapors of several essential oils showed antibacterial effects against some pathogenic bacteria and some spoilage of bacteria on glass [3]. An increased adhesion of hydrophilic bacteria (*Staphylococci* and *Streptococci*) was observed on substrates coated with bovine serum albumin (BSA) [4]. The adhesion of *Staphylococcus aureus* is independent of surface roughness when its size is a magnitude lower than microbial size [5]. *E. coli*, *P. aeruginosa,* and *S. aureus* present significantly different patterns of attachment on glass, but they all exhibit a greater propensity for adhesion to the “nano-smooth” surface of glass [6]. The retention patterns of five different marine bacteria after attachment to two types of glass surfaces, as-received and chemically etched, were investigated, and amplified bacterial attachment was observed on chemically etched, nano-smoother glass surfaces. Furthermore, enhanced bacterial attachment was accompanied by elevated levels of secreted EPSs [7]. The results of the above representative studies regarding bacterial adhesion, inactivation, and biofilm formation on glass contain some contradictions, suggesting that the effects of glass surface properties on bacterial adhesion, inactivation, and biofilm formation are complex phenomena. To elucidate the cause of these contradictions, it is necessary to understand the process through which biofilms attach to glass surfaces and the early stages of biofilm formation. The in situ three-dimensional observation of the early stages of biofilm formation in nanoscale water may help elucidate these processes.

An important analytical technique for understanding this is the in situ observation of how biofilms grow, dissolve, and change their morphology on various substrates in water. Several observation devices can be used for this purpose. Optical microscopes have insufficient resolution, and observing the detailed structure of biofilms using them is difficult, while electron microscopes cannot capture the original structure containing 70–90% water. Atomic force microscopes are suitable for observing the structure of bacteria and biofilms on which a small amount of EPSs has been produced, but when this amount exceeds a certain threshold, interaction with the cantilever probe may deform the soft biofilm, and it is difficult to say whether the shape of the biofilm, including EPSs, can be accurately observed and evaluated this way. Confocal laser scanning microscopy (CLSM), which is generally used for the nanoscale observation of biofilms in water in situ, is an excellent microscope that can visualize the arrangement of bacteria using appropriate staining and reveal the details of the biofilm structure [8,9,10,11,12,13,14,15,16,17,18,19,20]. However, depending on the substrate and observation conditions, problems such as unclear substrate position and the vertical resolution being lower than the horizontal resolution may occur.

A scanning ion conductance microscope (SICM) is a type of scanning probe microscope (SPM) that images the three-dimensional shape of the sample surface. It features a micro glass pipette electrode as a probe, which is a hollow micro glass pipette electrode with a tip opening diameter of about 100 nm. An Ag electrode covered with AgCl is inserted inside, and it is filled with an electrolyte. Meanwhile, another Ag/AgCl electrode is placed as a counter electrode in the electrolyte in which the sample is immersed, and an ion current is generated when a voltage is applied between the two electrodes. The SICM is commonly used to observe cells [21,22,23,24,25,26] or bacteria [27,28]. In a previous study, the authors applied this microscope to biofilms grown to a moderate degree on glass or transparent plastic and successfully observed the morphology of a biofilm formed by airborne resident bacteria in artificial seawater, as well as a single bacterial biofilm in phosphate buffer solution (PBS) formed by marine bacteria (*Aliivibrio fischeri*) [29]. However, when using SICM alone, only the shape of the sample surface can be obtained, and it is unclear whether what is observed is a bacterium or something else. There is also another disadvantage: it is impossible to observe parts that do not change the ion current. On the other hand, the aforementioned CLSM allows the user to observe the internal structure, such as the arrangement of bacteria and proteins, and it is expected that using SICM will overcome the disadvantages of CLSM, such as the difficulties in observing the substrate surface, the low resolution in the vertical direction, and the inability to measure non-fluorescent parts. In other words, by observing the same location with both SICM and CLSM, it is possible to make use of the advantages of each microscope while also overcoming their disadvantages.

In this study, we confirmed whether SICM could be used to observe several biofilms on glass at different growth levels; we then observed the same locations with SICM and CLSM and compared the results to study the morphology and three-dimensional structure of the biofilms. In addition, for biofilm observations using conventional SICM, a certain threshold value for the decrease in the ion current was considered to indicate the surface. In this study, we attempt to elucidate the internal structure of the biofilm by continuously measuring the decrease in the ion current at each probe depth (z position) and each observation point (xy position).

Glass was chosen as the substrate for same-site measurements using CLSM and SICM for two reasons: The first is that, as mentioned above, the efficiency of removing food poisoning bacteria is known to differ between glass substrates subjected to different sterilization treatments, and the establishment of this observation technique may shed light on these mechanisms. The second reason is that a transparent sample is desirable for same-site observations because it is easy to align the sample using an inverted microscope; thus, we decided to use glass, a transparent material. The reasons for using marine bacteria (*Aliivibrio fischeri*) in these observations are as follows: First, *Aliivibrio fischeri* has long been known as a biofilm-forming bacterium, and many studies [30,31,32,33,34,35] have already been conducted on the biofilms formed by *Aliivibrio fischeri* on glass substrates. In these studies, the detailed mechanism of biofilm formation has been clarified in some areas but not in others. In order to clarify the latter in the future, the observation target in this paper was set to biofilms formed by *Aliivibrio fischeri* on glass substrates. Second, the most suitable electrolyte for SICM observations is phosphate buffer solution, and marine bacteria produce a sufficient amount of biofilm for observation in this environment.

## 2. Materials and Methods

### 2.1. Biofilm Formation

A colony of marine bacteria (*Aliivibrio fischeri* JCM18803, RIKEN) on an agar medium (Difco^TM^ Marine Agar 2216, Becton, Dickinson and Company, Franklin Lakes, NJ, USA) was placed in a test tube and then cultured in liquid marine medium (Difco^TM^ Marine Broth 2216, Becton, Dickinson and Company, Franklin Lakes, NJ, USA) for 2 days at 22 °C. The mixture was diluted 32-fold with PBS. A glass-bottom dish (Glass Bottom Culture Dish GBCD15, Violamo, Osaka, Japan) and a gridded glass slide (GC1310 with grid, 13φ, Matsunami Glass Industry Co., Ltd., Osaka, Japan) were immersed in the mixture and left to stand at 22 °C for 2 days to generate a biofilm.

### 2.2. Fixation and Staining of Biofilms for Confocal Laser Scanning Microscopy (CLSM) and CLSM Observations

The biofilm was fixed for observation with multiple microscopes and stained for observation with a confocal laser scanning microscope. After biofilm generation, the culture medium was removed, and the biofilm was fixed for 1 h using PBS containing 4% glutaraldehyde. After fixation, the specimens were stained with crystal violet (CV) [36] solution for 1 h and then with DAPI (4′,6-Diamidino-2-phenylindole, dihydrochloride, solution, Bacstain-DAPI solution) [37] for 5 to 10 min. The CV solution was used to stain negatively charged areas such as cell membranes, and DAPI was used to stain bacterial nucleic acid (DNA). Fluorescence images of CV and DAPI were obtained by irradiating the specimens with diode lasers with wavelengths of 561 nm and 405 nm, respectively. The maximum fluorescence wavelengths of the CV solution and DAPI were 580 nm and 461 nm, respectively, and they were observed simultaneously using CLSM (LSM 980 with Airyscan 2, ZEISS, Oberkochen, Germany) by introducing appropriate wavelength filters into the laser receiving section. A three-dimensional image was obtained from 166 images taken at intervals of approximately 130 nm in the Z direction.

### 2.3. Scanning Ion Conductance Microscopy (SICM) Observations

**Figure 2:**
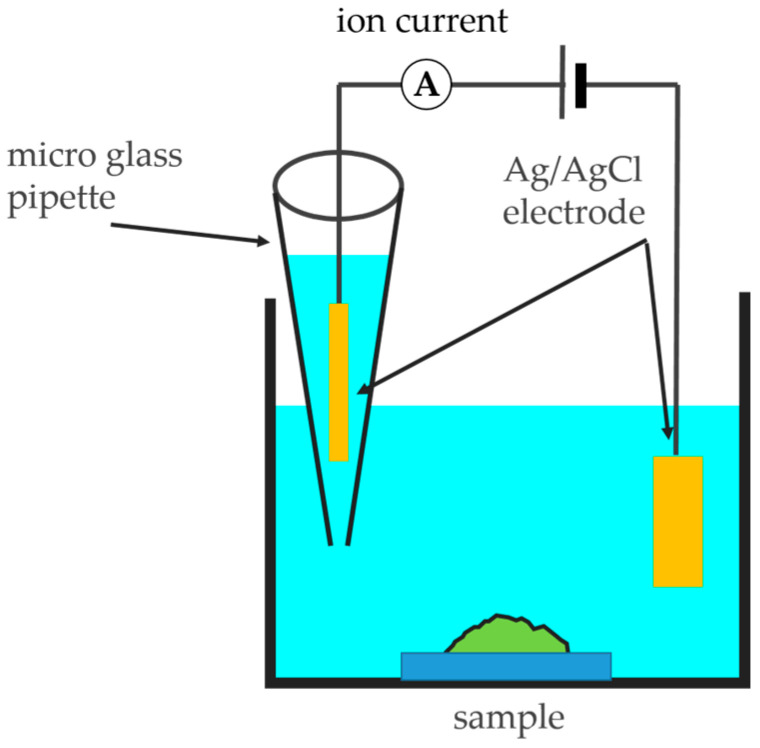
Outline of the SICM.

Figure 2 shows a schematic diagram of the SICM setup, which was made in-house [23,26]. In this study, the biofilm shape was imaged using the hopping mode (backstep mode). In this mode, at each scanning point, the tip is first raised to a position sufficiently far away from the sample. Then, the probe approaches the sample; it is blocked by the glass substrate or biofilm, causing the ion current to decrease. A threshold is set for the amount of decrease in the ion current, and the point where the ion current has decreased to the threshold is considered to be the glass substrate or biofilm surface. The probe is then moved away from the sample. By repeating the above process for each pixel, the biofilm’s shape can be obtained. As this process is repeated at each scanning point, the glass substrate and the biofilm formed thereon can be imaged. Although it has the disadvantage of taking a long time (about 0.1–8 h) to scan the sample, this observation mode is suitable for soft samples with severe unevenness; so, it is mainly used for observing biological samples such as cells. This mode was used for biofilm observation in this study. The scanning time depends on the height of the biofilm; obtaining one image of 32 × 32, 128 × 128, or 256 × 256 pixels required about 4–7, 60–120, or 240–480 min, respectively. The ion current threshold value for the ion current was mainly set to 0.8–1.0%. When the tip opening diameter of the micro glass pipette electrode is about 100 nm, the resolution in the horizontal direction (XY direction) is about 50 nm, which is half the opening diameter of the micro glass pipette. As mentioned above, SICM is a technique for observing surface shapes, and the ion current threshold value is generally set to 0.8–1.0%. However, in the second half of this study, the ion current threshold is set to 2.0%, which is higher than that used for surface observation. This is because after setting a higher threshold than usual, the probe can enter the inside of the biofilm. By continuously acquiring the ion current value during the approach, we attempted to observe not only the surface but also the inside of the biofilm.

## 3. Results and Discussion

### 3.1. Revealing Biofilm Morphology Using Scanning Ion Conductance Microscopy (SICM)

**Figure 3:**
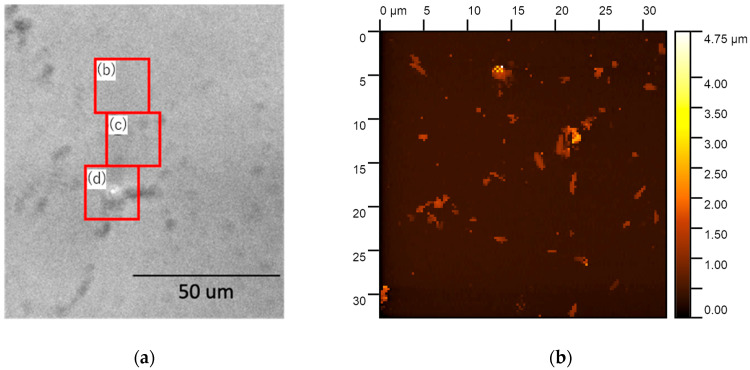

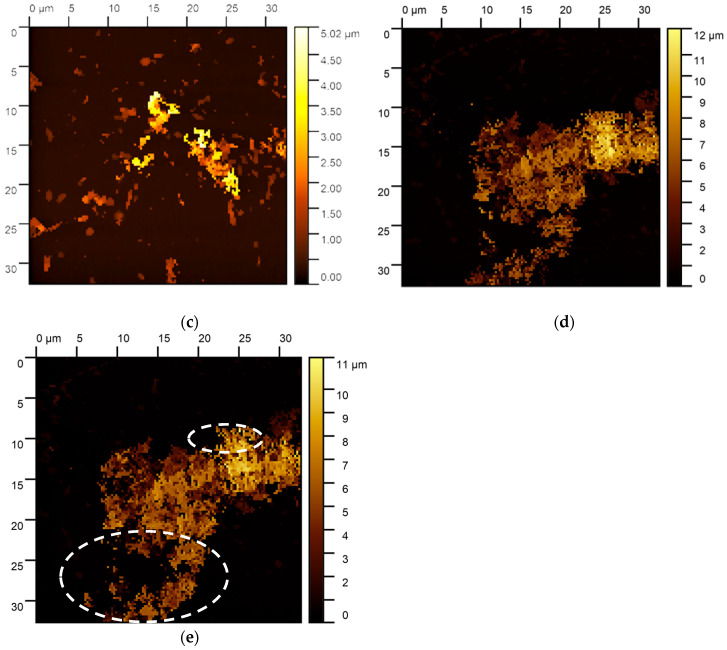
Three locations were observed using SICM: one where the biofilm was barely visible in the optical microscope image (**b**), one where a thin biofilm was visible (**c**), and one where a thick biofilm was visible (**d**). The optical microscope image is shown in (**a**). (**e**) shows the SICM image obtained at the same location for (**d**) after a rough scan (32 × 32 pixel) applied at negative bias. The images in (**b**–**e**) are obtained at 128 × 128 pixels. Comparing (**d**) and (**e**), the approximate shapes in the XY directions are the same, but parts that could not be detected in (**d**) are evident in (**e**), especially in the area surrounded by the white dashed line.

The biofilm formed on a glass-bottom dish was observed in situ using SICM in PBS at an ion current threshold value of 1% and a hopping distance of 12 μm. Before scanning with SICM, the scanning areas were determined using an optical microscope. The optical microscope image is shown in Figure 3a. Using the optical microscope, three locations, indicated by Figure 3b–d, were observed using SICM: in the first location, the biofilm was barely visible in the optical microscope image (Figure 3b); in the second, a thin biofilm was visible (Figure 3c); and in the third, a thick biofilm was visible (Figure 3d). The scanning range was all 33 × 33 μm, and the number of pixels of the images was 128 × 128 for all. The height is indicated by shades of yellow, with lighter areas being higher and darker areas being lower. In Figure 3b, bacteria with a length of about 2 μm and with a width and height of about 1 μm attached to the substrate surface can be observed, with almost no visible biofilm-like aggregates. In Figure 3c, the bacteria have aggregated, and a biofilm in the early stages of formation can be observed. The height of the biofilm is approximately 5 μm. In Figure 3d, a biofilm about 12 μm in height can be observed. As described above, it was found that a variety of biofilms, from those in the early stages of formation to thick biofilms, can be observed with SICM.

In the above observations, a positive potential was applied to the pipette electrode (positive bias). To clarify the effect of pipette electrode bias on the image, immediately after the observation in Figure 3d, negative potential was applied to the pipette electrode (negative bias), and the same area observed in Figure 3d was roughly scanned at 32 × 32 pixels; then, positive potential was applied to the pipette electrode, and the same area, at 128 × 128 pixels, was observed again. The results are shown in Figure 3e. Comparing Figure 3d,e, the approximate shapes in the XY directions are the same, but parts that could not be detected in Figure 3d are evident in Figure 3e, especially in the area surrounded by the white dashed line. Therefore, it was found that changing the bias once resulted in a clearer image. However, when comparing the biofilm height in Figure 3d,e, the height in the former figure is about 1 μm less than that in the latter. It is likely that some of the biofilm was lost due to the reverse-biased scanning micro glass pipette.

These experiments illustrate that scanning the micro glass pipette with a reverse bias before observing it with a positive bias is effective for capturing clear images. However, care should be taken, as there is a possibility that part of the biofilm may detach from the substrate. In order to prevent changes in the measurement, it is necessary to reconsider the ion current threshold, for example, by setting it to 0.8% or less.

### 3.2. Revealing the Biofilm Structure Using Scanning Ion Conductance Microscopy (SICM) Combined with Confocal Laser Scanning Microscopy (CLSM)

**Figure 4:**
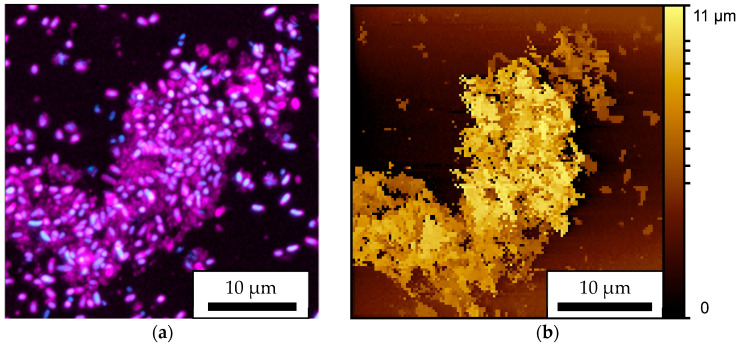
Biofilms observed using (**a**) CLSM and (**b**) SICM. The ion current threshold value for SICM observation was set to 1.0%.

Biofilms formed on a gridded slide glass were chemically fixed and stained with CV solution and DAPI. The same areas were observed using SICM and CLSM. The observation results are shown in Figure 4. Figure 4a is a CLSM image with a scanning range of 35 × 35 μm and a pixel count of 1007 × 1007 pixels. The areas where DAPI fluoresced are shown in blue, and the areas where CV fluoresced are shown in red; these fluorescence images are superimposed. Figure 4b is an SICM image with a scanning range of 33 × 33 μm and a pixel count of 128 × 128 pixels. Height is indicated by shades of yellow, with lighter areas being higher and darker areas being lower. The difference in height between the darkest and brightest areas is approximately 11 µm.

In this experiment, the ion current threshold value for SICM observation was 1.0%. Here, the threshold is an index of how much of the ion current is attenuated to determine the surface; the larger the value, the stronger the pressure. Biofilms of almost the same area and shape were observed.

**Figure 5:**
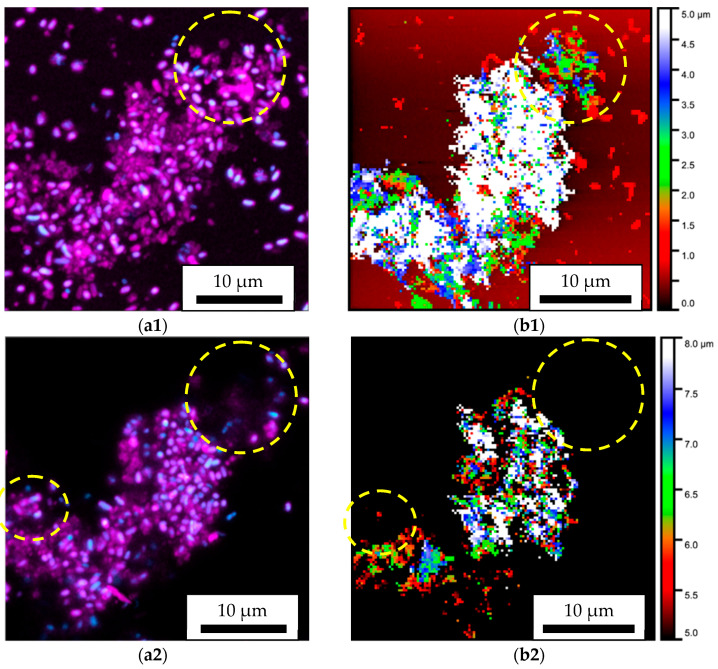

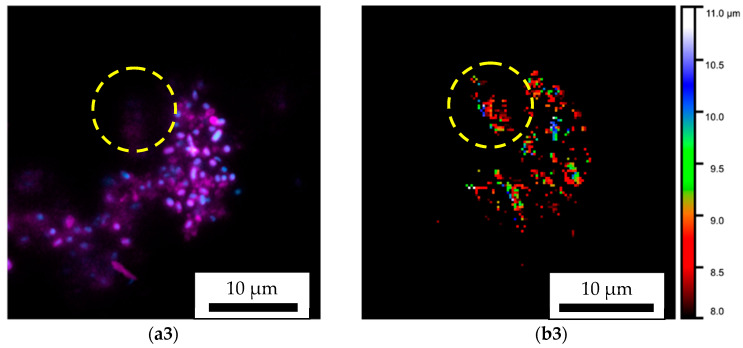
Biofilms observed using CLSM at (**a1**) 0–5 µm, (**a2**) 5–8 µm, and (**a3**) 8–11 µm from the substrate and those observed using SICM at (**b1**) 0–5 µm, (**b2**) 5–8 µm, and (**b3**) 8–11 µm from the substrate. Comparing (**a1**) and (**b1**), the shape of the biofilm and the arrangement of the bacteria are consistent. Biofilm in the part surrounded by a dashed yellow circle on the upper right is not detected in either (**a2**) or (**b2**). The areas within the dashed yellow circles on the left in (**a2**) and (**b2**) are fluorescent in CLSM but cannot be detected using SICM. Comparing (**a3**) and (**b3**), the part surrounded by a dashed yellow circle can be detected using SICM but does not fluoresce with CLSM.

**Figure 6:**
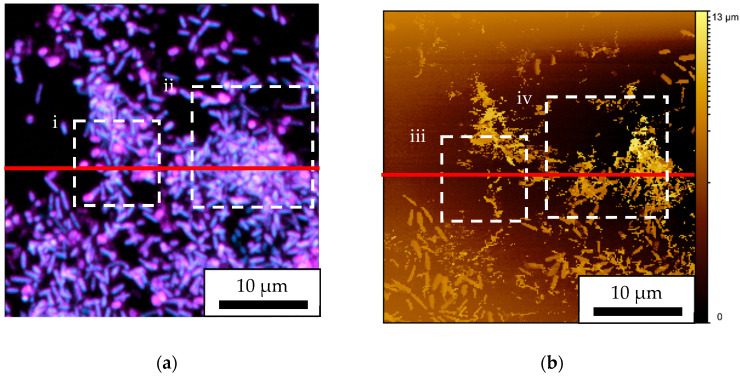
Biofilms observed two-dimensionally using (**a**) CLSM and (**b**) SICM. The ion current threshold value for SICM observation was set to 2.0%. Red line corresponds to ZX plane in Figure 7. Dashed white square (**i**–**iv**) corresponds to the location where the XY plane was observed in Figure 7(**ai**,**aii**,**biii**,**biv**).

In addition, to compare the height direction, the height of the area observed in Figure 4 was divided into three parts, with the result displayed in Figure 5. Figure 5(a1–a3) show CLSM images, and Figure 5(b1–b3) show SICM images. For CLSM images, the areas where DAPI fluoresced are shown in blue, and the areas where CV fluoresced are shown in red; these fluorescence images are superimposed. DAPI binds strongly to DNA and is therefore bound to cell nuclei and eDNA. CV is also used to quantify biofilms and is therefore thought to stain cell walls and EPSs. However, in these experiments, the fluorescence intensity of CV bound to EPSs was much weaker than that of CV bound to cell walls. In SICM images, height is indicated by rainbow color because of the improvement in resolution in the Z direction. In both CLSM and SICM images, the 3D observation results are cut out and displayed on a plane (XY plane) perpendicular to the normal direction of the substrate (Z direction) and are displayed in three parts: about Figure 5(a1,b1) 0–5 μm, Figure 5(a2,b2) 5–8 μm, and Figure 5(a3,b3) 8–11 μm from the substrate. In Figure 5(b1), white corresponds to Z > 5 μm and black to Z < 0 μm. In Figure 5(b2), white corresponds to Z > 8 μm and black to Z < 5 μm. Finally, in Figure 5(b3), white corresponds to Z > 11 μm and black to Z < 8 μm. Comparing Figure 5(a1,b1), the shape of the biofilm and the arrangement of the bacteria are consistent. In addition, the biofilm in the part surrounded by a dashed yellow circle on the upper right is not detected in either Figure 5(a2) or Figure 5(b2). This also shows that the height direction is consistent. However, comparing Figure 5(a3,b3), the part surrounded by a dashed yellow circle can be detected using SICM but does not fluoresce with CLSM. This suggests that there is something in this non-fluorescent area that cannot be stained with CV or DAPI. We believe that this region corresponds to the EPSs that do not bind to DAPI or CV. In addition, the areas within the dashed yellow circles on the left in Figure 5(a2,b2) are fluorescent in CLSM but cannot be detected using SICM. To clarify the cause of this, further observations were made in the height direction. Until this point, the probe had been moved sufficiently far away from the sample and swung down until the threshold value (1.0%) for the ion current was reached. However, in subsequent experiments, the threshold value for the ion current was set to 2.0%, which is stronger than that used in the conventional method, and the change in the ion current during the swing down was constantly recorded, allowing for observation even when the surface was far away or the micro glass pipette penetrated slightly into the biofilm. The XY observation image is shown in Figure 6.

The CLSM two-dimensional image in Figure 6a has a scanning range of 35 × 35 μm and a pixel count of 566 × 566 pixels, while the SICM two-dimensional image in Figure 6b has a scanning range of 35 × 35 μm and a pixel count of 256 × 256 pixels. The ion current threshold value for SICM observation was set to 2.0%. Evidently, the same area was observed. However, there were some areas where the observed height of biofilm was lower with SICM than with CLSM, and the accurate morphology could not be detected. We thought that this was due to the ion current threshold value of 2.0% being too large, which caused the pipette to be pressed too hard. Therefore, we decided to cut out the image in the ZX plane and perform a detailed analysis.

**Figure 7:**
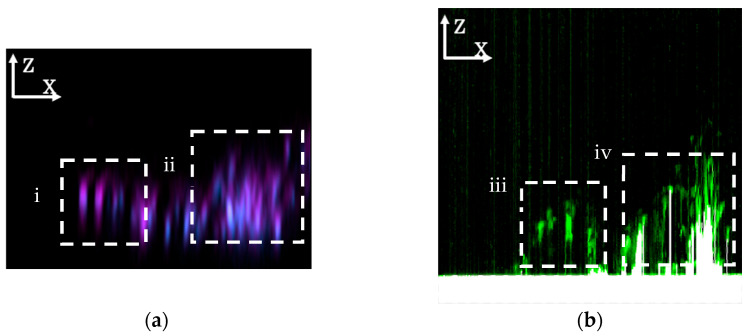
ZX plane images of biofilms observed using (**a**) CLSM and (**b**) SICM. The threshold value for the ion current for SICM observation was set to 2.0%. When the images in (**i**) and (**iii**) are considered together, a location where a large amount of ionic current can flow between the bacteria and the substrate can be detected. When the images in (**ii**) and (**iv**) are interpreted together, a place where a smaller amount of ionic current can flow between the bacteria and the substrate can be detected.

Figure 7 shows the ZX plane on the red line in the XY image (Figure 6). In the SICM ZX plane (Figure 7b), areas where no attenuation of the ion current is observed are shown in black, those where the attenuation reaches the ion current threshold value and is recognized as the surface are shown in white, and those where the ion current is attenuated but has not reached the threshold value (2.0%) are shown in green. Green areas are not shown in the XY image. In Figure 6b, green areas appear not to be detected in the dashed white square iii in the SICM XY image, but the ion current is evidently attenuated from the ZX plane (dashed white square iii in Figure 7b). Furthermore, fluorescence is observed in the same area in the CLSM ZX plane (Figure 7a). Based on this, it is thought that the three-dimensional structure of the biofilm could be accurately detected with CLSM in Figure 5(a2,b2) but not with SICM because the ion current threshold value of 1.0% was insufficient; however, we believe that this structure can be detected through SICM by observing the attenuation of the ion current in the height direction via ZXY observation.

In addition, when comparing the dashed white squares (i and ii) in Figure 7a, there is no significant difference in the way the fluorescence is observed, but when comparing the dashed white squares (iii and iv) in Figure 7b, the ease with which the ion current passes is clearly different. When the images in Figure 7(ai) and Figure 7(biii) are considered together, a location where a large amount of ionic current can flow between the bacteria and the substrate can be detected. Therefore, it can be seen that the EPSs on the underside of these bacteria easily conduct ionic currents. On the other hand, when the images in Figure 7(aii) and Figure 7(biv) are interpreted together, a place where a smaller amount of ionic current can flow between the bacteria and the substrate can be detected. Therefore, it appears that the EPSs on the underside of these bacteria do not conduct ionic currents well. SICM also provided insight into the internal structure of a biofilm when the actual threshold value was set to be higher than what is optimal for observing surface morphology. It is known that when food comes into contact with a surface such as glass, surface treatment affects the formation behavior and removal efficiency of bacteria and biofilms [2,3]; the observation method we have developed may help elucidate these mechanisms. Recent research [35] has revealed that calcium signaling controls the early stage of biofilm formation and dispersion in *Aliivibrio fischeri*. By simultaneously observing with SICM and CLSM, we believe that if we can visualize the three-dimensional morphology of the biofilm, the arrangement of bacteria within the biofilm, and the difference in local ion conductivity within the biofilm, we will be able to obtain more detailed insight into biofilm formation and dispersion.

## 4. Conclusions

In conclusion, this study illustrates that both scanning ion conductance microscopy (SICM) and confocal laser scanning microscopy (CLSM) are valuable techniques for observing biofilm formation, providing complementary insights into the morphology and structure of biofilms. SICM, with its high-resolution 3D imaging capability, allows for detailed observations of biofilm height and topography, enabling the identification of biofilms at various stages of development, from early-stage aggregates to thicker structures. The impact of adjusting the threshold value for the ion current and bias during SICM observations was significant. By changing the bias, clearer images were obtained; however, some biofilm material appeared to be displaced or removed due to the reverse bias, which highlights the importance of carefully selecting the measurement parameters to avoid altering the biofilm during observation.

Additionally, we were the first in the world to successfully perform in situ 3D analysis of the same location on a biofilm sample in liquid using both SICM and CLSM. When comparing SICM and CLSM images of the same biofilm samples, each technique revealed different aspects of the biofilm’s structure. SICM was able to reveal detailed structures of biofilms that could not be captured by CLSM because they are not stained. While SICM provided detailed topographical information, CLSM offered fluorescence-based insights that highlighted areas of the biofilm that were not detectable using SICM, such as regions where the biofilm exhibited fluorescence but had low ion current attenuation. This discrepancy was attributed to the ion current threshold value for SICM observation, which, when set too high, may result in some regions of the biofilm being missed. By adjusting the threshold value for the ion current and conducting ZXY observations, it may be possible to detect areas that were previously undetected, improving the overall accuracy of SICM-based biofilm three-dimensional imaging. In addition, SICM can also visualize the local ionic conductivity of biofilms, which is expected to provide insight into the effects of various ions that affect the properties of biofilms. As described above, by combining SICM and CLSM to observe the same location of a biofilm, it is possible to take advantage of the strengths of each and cover up their weaknesses, thereby enabling us to obtain more information than could be obtained from either microscope alone.

## Data Availability

The original contributions presented in this study are included in the article. Further inquiries can be directed to the corresponding author.
